# Effect of blood lead levels on periodontitis in American adults: a cross-sectional analysis from the national health and nutrition examination survey

**DOI:** 10.1186/s12903-024-04068-1

**Published:** 2024-03-21

**Authors:** Tangye Dai, Qun Dai

**Affiliations:** 1 Nanchang University Affiliated Stomatological Hospital, Jiangxi Province, China; 2 Hangzhou stomatological hospital, Hangzhou, Zhejiang, China

**Keywords:** Periodontitis, Blood lead, Heavy metal, NHANES

## Abstract

**Background:**

The primary objective of this study was to assess the impact of blood lead levels on the development and progression of periodontitis.

**Methods:**

This study included 8600 participants from the National Nutrition and Health Examination Survey conducted the United States between 2009 and 2014. The exposure variable was the blood lead level, while the outcome variable was periodontitis. To evaluate the relationship between the blood lead level and periodontitis, a multivariate logistic regression model was used.

**Results:**

A positive association was observed between blood lead levels and the risk of periodontitis in Model 1 (OR = 7.04, 95% CI = 5.95–8.31). After adjusting for age (continuous), sex, ethnicity, and BMI (continuous) in Model 2, the significant association between blood lead levels and periodontitis risk remained evident (OR = 3.06, 95% CI: 2.54–3.70). Consequently, even after comprehensive adjustment for potential confounding factors in Model 3, the robust association between blood lead levels and periodontitis risk persisted (OR = 2.08, 95% CI = 1.67–2.60). When considering the serum lead concentration as a categorical variable and after adjusting for potential confounders in Model 3, we observed that the odds ratios (ORs) of periodontitis in the T2 (0.94 µg/dL-1.60 µg/dL) and T3 (lead ≥ 1.60 µg/dL) groups increased from 1.27 (OR = 1.27, 95% CI: 1.11–1.44) to 1.57 (OR = 1.57, 95% CI: 1.36–1.81) compared to T1 group. Subgroup analysis revealed no effect modifiers.

**Conclusions:**

Our main findings suggest that there is no safe range of blood lead levels regarding periodontitis risk and that increasing blood lead levels can significantly increase the prevalence of periodontitis.

## Introduction

Periodontitis is a chronic infection of gram-negative tooth-supporting tissue and is widely recognized as one of the most prevalent chronic infections in humans[1]. According to relevant research, the prevalence of periodontitis in adults ranges from 40–72.4% [2–5], and periodontitis is widely recognized as a significant public health concern in many countries. Chronic inflammation of periodontal tissue is associated with an imbalance in plaque biofilms, resulting in progressive destruction of the supporting bone and subsequent tooth loss. This represents the fundamental pathological characteristic of periodontitis [6, 7]. Periodontitis not only induces local oral damage but also exacerbates the incidence of chronic noncommunicable diseases such as diabetes [8, 9], cardiovascular disease [10–13], and rheumatoid arthritis [14–16]. Although the primary risk factor for periodontitis is the accumulation of bacterial plaque (microbial biofilms) in the supragingival and subgingival areas [17, 18], it has been reported that individual lifestyle factors [19–21], environmental exposure to heavy metals [22, 23], socioeconomic status [24, 25], and other factors exert varying effects on the development and progression of periodontitis.

Lead is a naturally occurring metal and one of the earliest metals utilized in human history, making it the most readily available heavy metal in the environment. The majority of lead exposure is attributed to anthropogenic activities, including the combustion of fossil fuels (including the historical usage of leaded gasoline), specific industrial facilities, and the previous utilization of lead-based paint and tobacco within residential settings [26, 27]. It is widely accepted that the presence of heavy metals, such as lead, in aquatic environments can result in the accumulation of excessive toxic substances in aquatic organisms through water pollution; human consumption of fish contaminated with lead may cause continuous bioaccumulation of heavy metals in the bloodstream [28]. The presence of heavy metal pollutants can have detrimental impacts on human health. Furthermore, lead strongly affects mature bones in the human body. Calcified tissues, including bones and teeth, serve as the primary reservoirs for lead accumulation in both adults and children, with bone lead accounting for more than 90% of the total lead burden in adults [29, 30].Once lead is incorporated into the crystalline structure of bone, it remains a quiescent structural component within the bone matrix until osteoclast activity triggers its release into circulation [31, 32]. Therefore, we hypothesized that lead exposure and serum lead levels may serve as modifiable risk factors for periodontitis. Despite the gradual decrease in blood lead levels among American adults over the past few decades, the safe range of blood lead levels regarding periodontitis risk has not been determined.

Therefore, to address the research gap in this field, we utilized the National Health and Nutrition Examination Survey (NHANES) to empirically examine our research hypothesis, specifically assessing the impact of blood lead levels on the development and progression of periodontitis.

## Methods

### Study design and population

The National Health and Nutrition Examination Survey (NHANES) adheres to the STROBE guidelines [33]. The NHANES is a biennial nationwide survey that employs a stratified, multistage, and probabilistic sampling design to investigate and gather information on the health and nutritional status of the noninstitutionalized population in the United States. The data collection methods included questionnaires, interviews, and physical examinations. Detailed information regarding the survey’s methodology and design can be accessed through the following link: https://www.cdc.gov/nchs/nhanes/index.htm. The research plan of the NHANES is endorsed by the Ethics Review Committee of the National Center for Health Statistics, with all participants providing written informed consent. This study included a total of 8928 participants from the NHANES who were aged older than 18 years and had complete periodontal examination data from 2009 to 2014. After excluding individuals (*n* = 328) with missing blood lead data, the final cross-sectional analysis included a sample of 8600 participants.

### Periodontitis

The periodontal disease inspection process was as follows: First, a comprehensive periodontal examination was conducted by the dental examiner for participants aged older than 30 years with at least one natural tooth. Second, survey reference inspectors performed training and calibration among all dental inspectors. A color-coded periodontal probe was used to measure probing depth (PD) and gingival recession at six sites for each tooth, with measurements taken every two millimeters. Professional inspectors reviewed the measurement results for all four quadrants and rounded them to the nearest millimeter. During data input, an algorithm automatically calculated the clinical attachment loss (AL), which represents the distance from the cementoenamel junction (CEJ) to the bottom of the probe pocket [34, 35]. According to the definition of periodontitis provided by the American Center for Disease Control and Prevention/American Academy of Periodontology (AAP), the subjects were categorized into four groups: no periodontitis, mild periodontitis, moderate periodontitis, and severe periodontitis. Specifically, these categories were defined as follows: [1] mild periodontitis: ≥2 interdental sites with attachment loss (AL) ≥ 3 mm and a probing depth (PD) ≥ 4 mm but < 5 mm (not on the same tooth) or 1 site with a PD ≥ 5 mm; [2] moderate periodontitis: two or more dental spaces with AL ≥ 4 mm but < 6 mm or two or more dental spaces with a PD ≥ 5 mm (not on the same tooth); and [3] severe periodontitis: ≥2 interdental space sites with AL ≥ 6 mm on different teeth or ≥ 1 interdental space site with a PD ≥ 5 mm; individuals who did not fall into any of these categories were considered free from periodontal disease36. In accordance with this definition, our outcome variable was directly defined as whether an individual was diagnosed with periodontitis.

### Blood lead and other measurements

The concentrations of heavy metals, including lead, cadmium, and mercury, in blood samples were determined using inductively coupled plasma‒mass spectrometry (ICP‒MS). Detailed information regarding the laboratory procedures employed by the NHANES has been previously documented [37, 38]. Furthermore, the reported test results adhered to the appropriate quality control and assurance protocols [37, 39].Additionally, we also collected data on the following potential covariates: first, data on demographic characteristics were collected, including age (continuous variable), sex (male or female), ethnicity (non-Hispanic White, non-Hispanic Black, Mexican American, other Hispanic or other races), and the poverty-income ratio (PIR) [40]. Second, in terms of lifestyle, individuals were categorized into three groups according to smoking status: never smokers (those who had smoked fewer than 100 cigarettes in their lifetime), current smokers (individuals who had smoked at least 100 cigarettes in their lifetime and continued to smoke), and former smokers (individuals who had smoked at least 100 cigarettes in their lifetime but had quit smoking) [41]. In addition, other covariates, such as BMI (continuous variable), glycosylated hemoglobin, total cholesterol (TC), high-density lipoprotein (HDL), and serum uric acid (SUA) levels, estimated glomerular filtration rate (eGFR), diabetes, hypertension and hyperlipidemia status, antihypertensive and hypoglycemic drug use, and history of drug use were included in the analysis. Participants were classified as having diabetes if they met one of the following criteria: [1] had a self-reported diagnosis of diabetes, [2] were currently using hypoglycemic agents, or [3] had a fasting blood glucose level ≥ 7.0 mmol/L [42]. Patients who exhibited an average systolic blood pressure (SBP) and diastolic blood pressure (DBP) ≥ 140/90 mmHg on three separate occasions or were taking antihypertensive medication were classified as having hypertension [43].

### Statistical analysis

The distribution of blood lead levels in our study was skewed; therefore, we performed a Log10 transformation (Lg lead) during the data analysis. The classification variables are expressed as percentages (n%), means with standard deviations for normally distributed continuous variables, and medians with interquartile ranges (IQRs, Q1-Q3) for nonnormally distributed continuous variables. The normally distributed data were analyzed by one-way ANOVA, the nonparametric nonnormally distributed data were tested by the Mann‒Whitney test, and the difference between the blood test results was calculated by the chi-square test for continuous variables and categorical variables. A multivariate logistic regression model was subsequently used to evaluate the association between blood lead levels and periodontitis risk while considering relevant factors. Model 1 was unadjusted. Model 2 was adjusted for age, sex, ethnicity, and BMI. Model 3 was adjusted for age, sex, ethnicity, BMI, PIR, current smoking status, glycated hemoglobin, TC, HDL, and SUA levels, eGFR, diabetes, hypertension, and hyperlipidemia status, antihypertensive drug use, lipoprotein-lowering drug use, glucose-lowering drug use, and cadmium and mercury levels. The potential dose‒response relationship between blood lead concentrations and the incidence of periodontitis was analyzed using a generalized additive model and fitting curve. Furthermore, the potential dose‒response relationship between blood lead levels and the incidence of periodontitis was examined using a generalized additive model and fitting curve analysis. Additionally, stratified analysis and cross-validation were performed on the following variables to assess their association with both blood lead levels and periodontitis incidence: sex, age, BMI, race, diabetes status, hypertension status, and hyperlipidemia status.

The final statistics and data analysis were conducted using R (R Foundation for Statistical Computing, http://www.r-project.org) and Empower (R) (X&Y Solutions, Inc.; www.empowerstats.com). A bilateral p value of less than 0.05 was considered to indicate statistical significance.

## Results

### Study participants and baseline characteristics

In this cross-sectional study of 8600 individuals, the mean age (standard deviation:SD) was found to be 51.99 ± 14.23 years. The proportion of males was 49.52% (4259), and the median serum lead concentration (interquartile range: IQR) was 1.23 (0.81–1.87). Table 1 presents the baseline characteristics of the participants categorized according to their serum lead levels. As indicated in Table 1, the highest serum lead group, T3 (lead level ≥ 1.60), exhibited the following characteristics: a greater proportion of male individuals, a greater proportion of older individuals, an increased representation of non-Hispanic black individuals, and predominantly current smokers. Compared to the group with lowest serum lead levels, the group with the highest serum lead levels exhibited a lower BMI, eGFR and PIR, and higher TC, HDL, and SUA levels, as well as elevated concentrations of mercury and chromium. Additionally, there was a higher incidence of hypertension and hyperlipidemia, increased utilization of antihypertensive drugs and lipoprotein-lowering drugs, and a lower usage rate of glucose-lowering drugs in the group with the highest lead levels. However, no statistically significant differences were observed in the glycated hemoglobin values or diabetes incidence among the three groups (*p* > 0.05).

**Table 1:** The demographic and clinical characteristics of the patients by tertiles of baseline lead

Characteristics	Lead, µg/dL			P value
	T1 (< 0.94)	T2 (0.94–1.60)	T3 (≥ 1.60)	
N	2865	2856	2879	
Males, N(%)	986 (34.42%)	1449 (50.74%)	1824 (63.36%)	< 0.001
Age, years	45.41 ± 12.66	53.35 ± 13.74	57.20 ± 13.65	< 0.001
Ethnicity				< 0.001
Non-Hispanic White,N(%)	1297 (45.27%)	1221 (42.75%)	1211 (42.06%)	
Non-Hispanic Black,N(%)	513 (17.91%)	574 (20.10%)	673 (23.38%)	
Mexican American,N(%)	417 (14.55%)	404 (14.15%)	427 (14.83%)	
Other Hispanic,N(%)	362 (12.64%)	300 (10.50%)	206 (7.16%)	
Other races,N(%)	276 (9.63%)	357 (12.50%)	362 (12.57%)	
BMI, kg/m$	30.67 ± 7.61	29.20 ± 6.30	28.02 ± 5.74	< 0.001
PIR	2.73 ± 1.65	2.71 ± 1.67	2.51 ± 1.65	< 0.001
Current smoking, N(%)	334 (11.66%)	509 (17.82%)	780 (27.11%)	< 0.001
glycated hemoglobin,%	5.79 ± 1.18	5.84 ± 1.09	5.81 ± 0.97	0.151
TC, mg/dL	194.05 ± 39.50	199.74 ± 41.54	202.13 ± 42.05	< 0.001
HDL, mg/dL	51.78 ± 14.91	52.78 ± 16.13	54.28 ± 17.11	< 0.001
SUA, mg/dL	5.13 ± 1.41	5.49 ± 1.40	5.72 ± 1.39	< 0.001
eGFR, mL/min/1.73 m2	99.00 ± 18.97	90.62 ± 19.72	85.89 ± 21.51	< 0.001
Mercury,medium (IQR), µg/L	0.82 (0.44–1.56)	1.02 (0.56–2.15)	1.07 (0.54–2.41)	< 0.001
Cadmium, medium (IQR), µg/L	0.26 (0.16–0.40)	0.34 (0.22–0.57)	0.45 (0.27–0.81)	< 0.001
hypertension, n (%)	1017 (35.50%)	1275 (44.66%)	1472 (51.13%)	< 0.001
hyperlipidemia, n (%)	2035 (71.03%)	2174 (76.12%)	2245 (77.98%)	< 0.001
diabetes, n (%)	438 (15.51%)	444 (15.59%)	439 (15.25%)	0.935
Antihypertensive drugs	726 (25.34%)	965 (33.79%)	1035 (35.95%)	< 0.001
Lipoprotein-lowering drugs	450 (15.71%)	634 (22.20%)	698 (24.24%)	< 0.001
Glucose-lowering drugs	343 (11.97%)	334 (11.69%)	280 (9.73%)	0.013

Abbreviations: BMI body mass index, PIR poverty income ratio, TC Total cholesterol, HDL high-density lipoprotein, SUA serum uric acid, eGFR estimated glomerular filtration rate

^$^BMI was calculated as the body weight in kilograms divided by the square of the height in meters

### Associations between blood lead levels and periodontitis

**Fig. 1:**
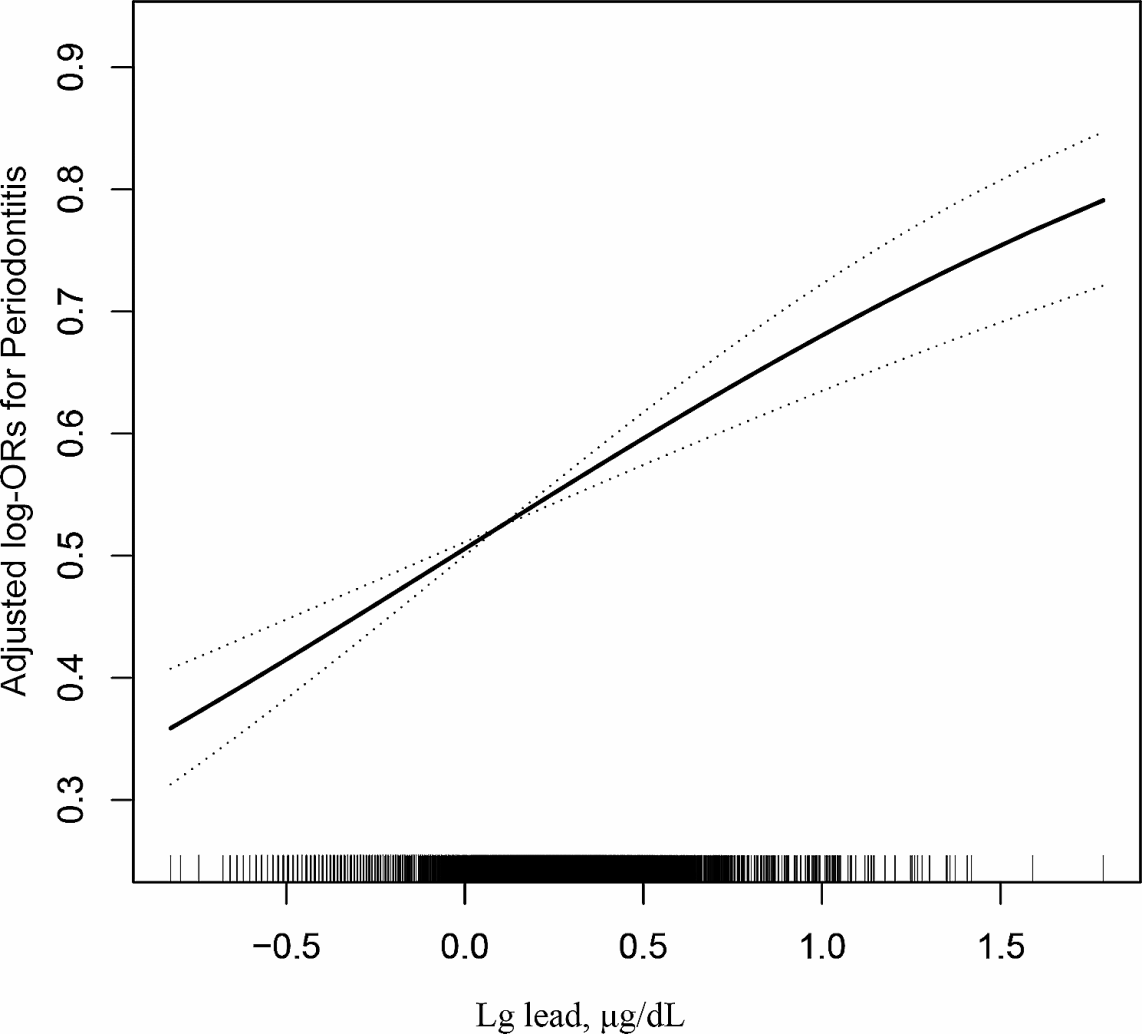
Association between blood lead levels with periodontitis. A linear association between blood lead levels with periodontitis was found (*P* < 0.05). The solid line and dashed line represent the estimated values and their corresponding 95% confidence interval. Adjustment factors included age, sex, ethnicity, BMI, PIR, current smoking, glycated hemoglobin, TC, HDL, SUA, eGFR, diabetes, hypertension, hyperlipidemia, antihypertensive drugs, lipoprotein-lowering drugs, glucose-lowering drugs, cadmium, mercury

As presented in Table 2, a positive association was observed between blood lead levels and the risk of periodontitis in Model 1 (OR = 7.04, 95%CI: 5.95–8.31, *p* < 0.0001). After adjusting for age (continuous), sex, ethnicity, and BMI (continuous) in Model 2, the significant association between blood lead levels and periodontitis remained evident (OR = 3.06, 95% CI: 2.54–3.70, *p* < 0.0001). Consequently, even after comprehensive adjustment for potential confounding factors in Model 3, a robust association persisted between blood lead levels and periodontitis (OR = 2.08, 95% CI:1.67–2.60, *p* < 0.0001). When considering the serum lead level as a categorical variable and after adjusting for potential confounders in Model 3, we observed that the odds ratio (OR) of periodontitis in the T2 (0.94 µg/dL-1.60 µg/dL) and T3 (lead ≥ 1.60 µg/dL) groups increased from 1.27 (OR = 1.27, 95% CI: 1.11–1.44, *p* = 0.0004) to 1.57 (OR = 1.57, 95% CI: 1.36–1.81, *p* < 0.0001) compared to participants in the T1 group, after adjusting for potential confounders in model 3. Furthermore, the *P* for trend < 0.0001 indicated a significant linear positive association between serum lead levels and periodontitis. The dose‒response relationship between blood lead levels and periodontitis risk according to the generalized additive model and fitting curve analysis is illustrated in Fig. 1, providing further evidence of a linear positive association between these factors.

**Table 2:** Relative odds of Periodontitis according to lead in different models

Lg lead	Events (%)	Periodontitis, OR (95%CI), P value		
		Model 1	Model 2	Model 3
Per 1unit increase	4542 (52.81%)	7.04 (5.95, 8.31) <0.0001	3.06 (2.54, 3.70) <0.0001	2.08 (1.67, 2.60) <0.0001
Tertiles				
T1 (< 0.94)	1071 (37.38%)	1	1	1
T2 (0.94–1.60)	1545 (54.10%)	1.97 (1.78, 2.19) <0.0001	1.40 (1.25, 1.58) <0.0001	1.27 (1.11, 1.44) 0.0004
T3 (≥ 1.60)	1926 (66.90%)	3.39 (3.04, 3.77) <0.0001	2.00 (1.77, 2.27) <0.0001	1.57 (1.36, 1.81) <0.0001
P for trend		< 0.0001	< 0.0001	< 0.0001

Model 1 was adjusted for none

Model 2 was adjusted for age, sex, ethnicity, BMI.

Model 3 was adjusted for age, sex, ethnicity, BMI, PIR, current smoking, glycated hemoglobin, TC, HDL, SUA, eGFR, diabetes, hypertension, hyperlipidemia, antihypertensive drugs, lipoprotein-lowering drugs, glucose-lowering drugs, cadmium, mercury

### Subgroup analyses

**Fig. 2:**
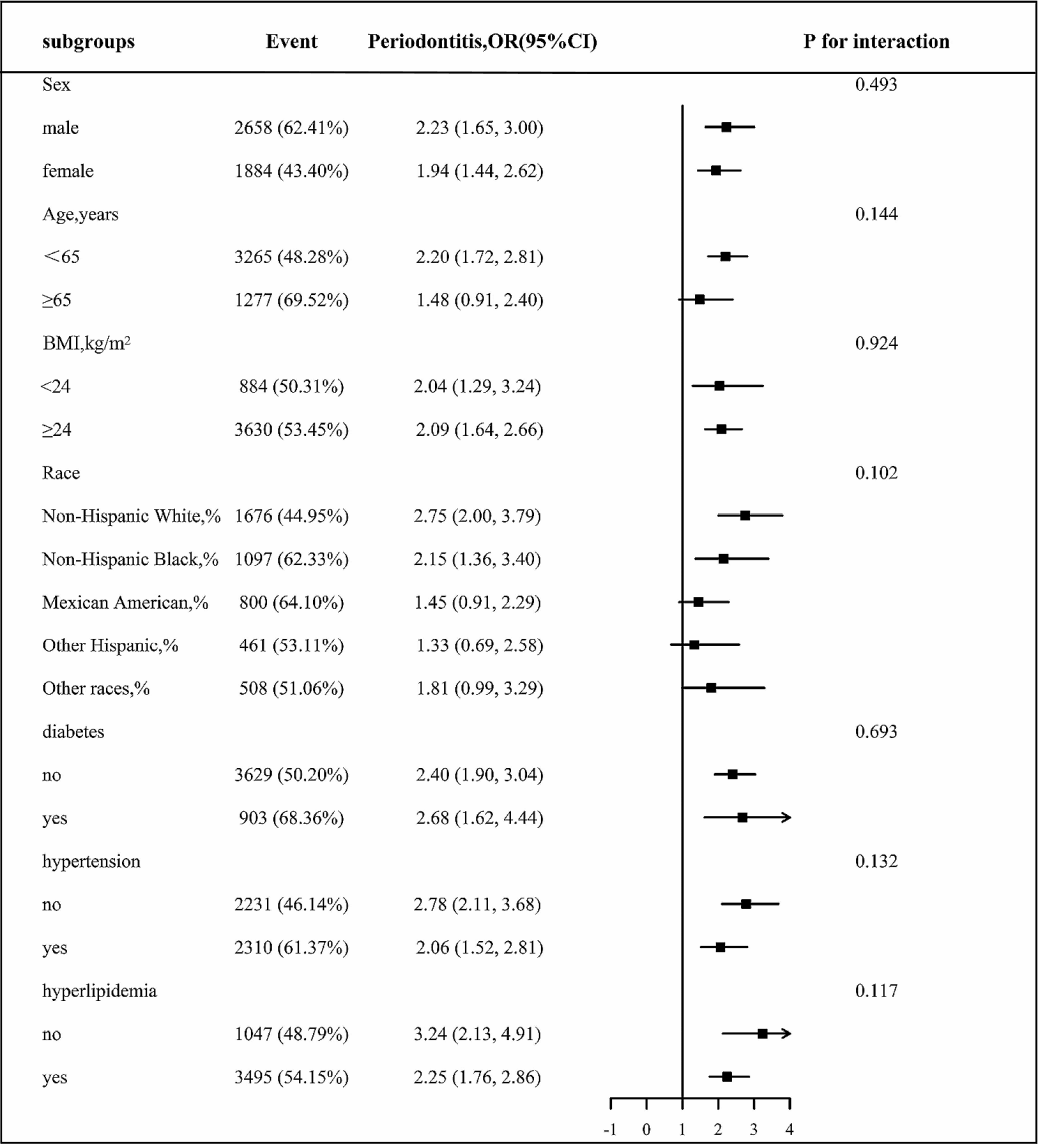
Stratified analyses by potential modifiers of the association between blood lead levels with periodontitis^**^ Each subgroup analysis adjusted for age, sex, ethnicity, BMI, PIR, current smoking, glycated hemoglobin, TC, HDL, SUA, eGFR, diabetes, hypertension, hyperlipidemia, antihypertensive drugs, lipoprotein-lowering drugs, glucose-lowering drugs, cadmium, mercury except for the stratifying variable

Figure 2 illustrates the outcomes of the association between periodontitis and blood lead levels across various subgroups. After adjustment for confounding variables, individuals who were male, were under 65 years old, had a BMI ≥ 24 kg/m2, were non-Hispanic white, had diabetes, were nonhypertensive, or were nonhyperlipidemic exhibited increased susceptibility to periodontitis in the presence of lead exposure; however, the interaction with blood lead levels was not statistically significant.

## Discussion

The present study investigated, for the first time, the association between blood lead levels and periodontitis in a large sample population from 2009 to 2014. Our main findings suggest that there is no safe range of blood lead levels regarding periodontitis risk, with an observed increase in disease incidence corresponding to higher blood lead concentrations. Furthermore, after we adjusted for confounding factors, including age, sex, ethnicity, BMI, PIR, current smoking status, glycated hemoglobin, TC, HDL, and SUA levels, eGFR, diabetes, hypertension, and hyperlipidemia status, antihypertensive drug use, lipoprotein-lowering drug use, glucose-lowering drug use, and cadmium and mercury levels, the aforementioned findings still retained statistical significance.

Currently, only one study has reported on the association between blood lead levels and oral diseases. Bruce et al. conducted a cross-sectional study involving 10,033 participants aged 20–69 years from the NHANES III (1988–1994) to investigate the relationship between blood lead levels and periodontal bone loss. The findings of this study revealed a significant positive association between elevated blood lead levels and an increased risk of periodontal bone loss, with greater susceptibility observed among current and former smokers [22]. In fact, the influx of lead into the bloodstream may be associated not only with regular bone turnover but also with periods of rapid bone metabolism. Studies have indicated that mineral deficiencies during pregnancy, during lactation, dietary intake, and in individuals with certain diseases (such as hyperthyroidism and osteoarthritis) can result in elevated blood lead levels [44, 45]. Furthermore, there have been reports suggesting an association between lead exposure and dental caries in children [46]. It appears that accelerated bone turnover or mineral loss contributes to an increase in blood lead levels; thus, initial assessment of body’s bone metabolism can be inferred from the level of blood lead concentration. Tooth loss and periodontal status are among the primary age-related conditions observed.

Many individuals with periodontal disease are often asymptomatic and are characterized by the progressive destruction of periodontal tissue over time, followed by a subsequent incubation period. The development of moderate and severe periodontal disease is characterized by irreversible alveolar bone loss, the continuous deepening of periodontal pockets, and increased clinical attachment loss. The formation of diseased periodontal pockets results from soft tissue inflammation and alveolar bone loss. When these pockets extend toward the apex of the tooth, the destruction of tooth attachment and supporting alveolar bone occurs, thereby increasing the risk of tooth loss. In posterior teeth that are severely affected, extensive attachment loss can result in destruction between root surfaces, leading to furcation involvement. The latest epidemiological research findings in the United States indicate that among adults aged 30–90 years, approximately 53% exhibit a minimum attachment loss of ≥ 3 mm for at least one tooth, while nearly 64% present a tooth pocket depth of ≥ 3 mm [47]. These observations collectively elucidate the detrimental impact of elevated blood lead levels on periodontal disease.

The advantage of this study lies in its pioneering investigation into the association between blood lead levels and periodontal disease risk within a large sample cohort of American adults. This cross-sectional study revealed that the incidence of periodontal disease increased with increasing blood lead levels. Subgroup analysis revealed no interactions between sex, age, BMI, race, complications and blood lead levels and periodontal disease risk; however, we found that increased blood lead levels led to an increased incidence of periodontal disease in men, individuals aged < 65 years, individuals with a BMI ≥ 24 kg/m2, non-Hispanic white individuals, individuals with diabetes, individuals without hypertension and individuals without hyperlipidemia. Because periodontal disease is an infectious disease causing a severe inflammatory response, compared with women, men are more likely to be exposed to cardiovascular risk factors, so their inflammatory response and oxidative stress are more significant. Overweight, obesity and diabetes also increase the risk of periodontal disease [48, 49]. Because hypertension and hyperlipidemia [50–53] are related to periodontal disease, these two diseases may conceal the independent effect of blood lead levels on periodontitis risk. The possible mechanism of periodontal disease caused by blood lead levels is as follows: lead is mostly deposited in teeth or calcium-rich places. With too much lead deposition, enamel is destroyed, teeth begin to fall out, and lead accumulation has toxic effects on dental nerves, which may lead to adverse reactions such as atrophy of dental nerves. In addition, blood lead can lead to an increase in the immune response [54] and the enhancement of immune cell activity, thus causing inflammation. Despite the gradual decrease in individuals’ blood lead levels in recent decades, the detrimental impact of elevated blood lead levels on periodontal disease risk persists, indicating that there is no safe threshold for blood lead levels; lower levels are preferable. In comprehensive assessments of oral health, monitoring clinical patients’ blood lead levels can effectively prevent the onset and progression of periodontal diseases.

However, certain limitations should be acknowledged when interpreting the findings of this study. First, due to its cross-sectional design, establishing a causal relationship between blood lead levels and periodontal disease risk in this study is challenging. Second, elevated blood lead levels may lead to periodontitis by changing the oral microbial flora, but we did not collect relevant data on oral microorganisms; therefore, we will further explore the relationships among these factors in future research to enrich the related fields. Finally, although our research benefited from a large sample size and the meticulous measurement of significant covariates (including smoke exposure), potential unmeasured or misreported variables could confound the observed association between blood lead levels and periodontal disease.

## Conclusions

With rapid societal development and escalating heavy metal pollution in the environment, the impact on overall health has transcended previous insignificant levels, thereby causing the increase in human blood lead levels due to environmental exposure to become a significant public health concern. Although knowledge regarding the influence of blood lead levels on oral health is limited, our research findings suggest that blood lead levels could serve as a crucial risk factor for adult periodontal disease. Prospective epidemiological studies and controlled animal experiments are imperative to validate and elucidate the underlying mechanisms involved.

## Data Availability

Publicly available datasets were analyzed in this study. This data can be found here: https://www.cdc.gov/nchs/nhanes/index.htm.
